# Side Effects after Second Dose of Covishield Vaccine among Healthcare Workers: A Descriptive Cross-sectional Study

**DOI:** 10.31729/jnma.6556

**Published:** 2021-06-30

**Authors:** Khilasa Pokharel, Bishwa Raj Dawadi, Anup Karki

**Affiliations:** 1 Department of Microbiology, Kathmandu Medical College and Teaching Hospital, Nepal; 2 Department of Emergency Medicine, Grande International Hospital, Nepal

**Keywords:** *dizziness*, *fatigue*, *pain*, *site*

## Abstract

**Introduction:**

COVID 19 vaccination will protect us from getting COVID-19. Some side effects are common which are signs that our body is building protection. This side effects will go away in a few days. The aim of this study is to find out the side effects seen among health care workers after second dose of covishield vaccination.

**Methods:**

This was a descriptive cross-sectional study conducted at a tertiary care centre from 22^nd^ April 2021 till 30^th^ April 2021. Ethical approval was revceived from Institutional Review Commitee. Convienient sampling was done. The second dose of covishield vaccine was administered 12 weeks after its first dose. The vaccine was administered intramuscularly into deltoid muscle. Statistical Package for the Social Sciences were used for analysis.

**Results:**

Out of 220 cases taken, 178 (80.90%) complained of pain at injection site after second dose of covidshield vaccine followed by 97 (44.09%) complaint of fatigue, 43 (19.54%) complaint of headache, 18 (8.18%) complaint of chills, 11 (5.00%)complaint of fever, 6 (2.72%) complaint of dizziness and 5 (2.27%) complaint of nausea.

**Conclusions:**

Pain at injection site, fatigue and headache were common side effects seen after second dose of Covishield vaccination.

## INTRODUCTION

The severe acute respiratory coronavirus-2 (SARS-Cov-2) was identified first in Wuhan city, Hubei Provience in China, at late 2019.^1^ A protective measure is required for herd immunity to SARS-Cov-2 infection for controlling COVID-19 pandemic.^2^

Essential aspect of any developing vaccine is to know the theoretical safety risks are identified and quantified against the potential benefits. Regarding the potential risk in case of COVID-19 vaccine development is whether the immune responses elicited by a vaccine could enhance SARS-Cov-2 acquistion or make disease worse when infection occurs after vaccination.^3^ As a preventive measure the Oxford-Astrazeneca's Covid-19 vaccine AZD 1222 was developed in Serum Institute of India as a Covishield vaccine.^4^ This vaccine was vectored with the chimpanzee adenovirus and it was found to have an average efficacy of 70.4% in a peer reviewed group.^5^ This vaccine has been donated to Nepal by Indian government and it can be stored in 2-8 degree Celsius.

The aim of this study is to know the side effect of this second dose of vaccine among healthcare workers.

## METHODS

The descriptive cross-sectional study was conducted at Kathmandu Medical College and Teaching Hospital between 20^th^ April 2021 till 10^th^ May 2021. Ethical approval was taken from Institutional Review Committee of Kathmandu Medical College and Teaching Hospital (Reference no: 0904202101). All the health care workers who have given consent were included in this study. Healthcare workers who has not given written consent and who are not vaccinated with second dose of Oxford-Astrazeneca's Covid-19 vaccine were excluded from the study in this study.

Sample size was calculated by using formaula,

$$\begin{array}{ll} \text{n} & \text{= }{\text{Z}}^{\text{2}}\times \text{p}\times \text{q}/{\text{e}}^{\text{2}}\\ & \text{= }{\left(1.96\right)}^{\text{2}}\times (0.138)\times (1-0.138)/{(\text{0.05)}}^{\text{2}}\\ & \text{= }182.79\\ & \approx 183 \end{array}$$

Where,

n = sample size
Z = 1.96 at 95% Confidence Interval
p = prevalence from the previous study, 13.8%^3^
q = 1-p
e = margin of error, 5%

The vaccine was administered after 12 weeks of first dose of covishield vaccine administration. It was administered Intramuscularly (IM) into deltoid muscle. Total 220 cases were included in this study. Health Care workers were enrolled in this study after receiving second dose of Covishield Vaccine by taking written informed consent. Everyone were given opened end question after 2 days to 7 days of vaccination. Statistical Package for the Social Sciences were used for analysis.

## RESULTS

**Figure 1:**
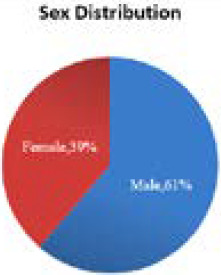
Gender wise distribution of cases.

Out of 220 cases who have taken second dose of covishield vaccine, 135 (61.36%) were male and 85 (38.63%) (Figure 1).

**Figure 2:**
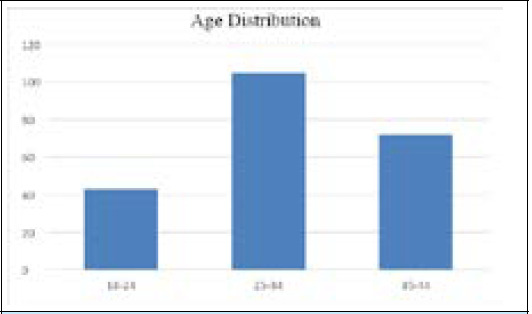
Distribution of cases on basis of age.

Among 220 cases, when we distributed the cases according to age 105 were between age group 25 to 34, 72 were between age 35 to 44 and and 43 were between age group 18 to 24 (Figure 2).

When we arrange the cases we found that, pain at injection site 178 (80.90%), fever 11 (5.00%), fatigue 97 (44.09%), headache 43 (19.54%), chills 18 (8.18%), diarrhoea 0 (0%), dizziness 6 (2.72%), and nausea 5 (2.27%) are the side effects complaint after second dose of covishield vaccination (Table 1).

**Table 1:** Side effect profile of vaccine.

	n (%)
Pain at injection site	178 (80.90)
Fever	11(5)
Fatigue	97 (44)
Headache	43(19)
Chills	18 (8)
Diarrhoea	0 (0 )
Dizziness	6 (2)
Nausea	5 (2)

## DISCUSSION

Covishield is the local version of Oxford-AstraZeneca COVID-19 vaccine. Serum institute of India, which is the largest vaccine manufacturer by volume, tied up with AstraZeneca to produce 1 billion doses of its COVID-19 vaccine.^6^ When we were looking for the side effects after second dose of covishield vaccine we found out that not all but some people were found to have side effect after second dose of covishield vaccine.

In this study, maximum number of health care workers 178 (80.90%) complaint of pain at injection site after second dose of covishield vaccine. Similarly, 97 (44.09%) complaint of fatigue, 43 (19.54%) complaint of headache, 18 (8.18%) complaint of chills, 11 (5.00%) complaint of fever, 6 (2.72%) complaint of dizziness and 5 (2.27%) complaint of nausea. This study was similar to the study reported before which shows pain, tenderness, warmth at the site of injection, fatigue, chills, headache, muscle pain, nausea, joint pain and feverishness as a very common side effect of covishield vaccine of which chills and nausea were reported as common side effect after second dose of vaccination.^7^

The limitation of the study is that we could not collect the data from other population who have received second dose of covishield vaccine.

## CONCLUSIONS

Vaccine have numerous positive effects including disease prevention. But there are some side effects felt after second dose of Covishield vaccine which mostly include pain at site of injection, fatigue and headache.
